# Private expenditures on healthcare: determinants, patterns and progressivity aspects

**DOI:** 10.1186/s13584-019-0356-y

**Published:** 2019-12-20

**Authors:** Leah Achdut

**Affiliations:** The Economic and Management Department, Ruppin Academic Center, Emek Hefer, Israel

**Keywords:** Health system finance, Public-private mixture, Socioeconomic inequalities, Concentration index, Decomposition, Equity in healthcare finance, Kakwani index

## Abstract

**Background:**

The mixture of public vs. private financing of the healthcare system has important fiscal and economic welfare implications. The consequences of this mixture for access to health services and for equity have become highly debated policy issues. In the first decade of 2000s, Israel experienced a continuous upward trend in the proportion of private financing, reaching a peak of 37–38%, which has subsequently leveled off. The 38% level is significantly higher than the 26% average across the Organization for Economic Co-operation and Development (OECD) countries.

**Main text:**

A recent paper by Tur-Sinai et al. in this journal shows that private spending by Israelis on health care is positively related to the income of individuals and to the socioeconomic status of their place of residence. This commentary draws attention to studies that integrate into one model both demand-side and supply-side determinants of private expenditures on healthcare. It also discusses inequity.

**Conclusions:**

Overall, the financing of national health expenditures in Israel is slightly regressive, but the progressivity of public financing is almost enough to offset the regressive effect of out-of-pocket payments and payments for voluntary complementary insurance.

## Background

In their paper on the determinants of private expenditures on healthcare in Israel, Aviad Tur-Sinai and his colleagues [7] focus on the direct and indirect effects of individuals’ socioeconomic and health characteristics on spending patterns, through the prism of single-person households. Given that the unit of monetary expenditure in traditional household expenditure surveys is the household, the choice to study only single-person households enables the authors to establish a direct relation between an individual’s state of health and its effect on spending patterns. The conceptual model proposed by the authors names income, socioeconomic status of place of residence (SES), and gender as the three predictors for health self-assessment, while four variables (the three mentioned above and health) serve as predictors of various types of private expenditures. The authors distinguish between voluntary health insurance payments and three types of out-of-pocket expenses and explore the relationships among the model’s variables separately for each of the following age groups: 20–29, 30–64, and 65 and over.

The picture that emerges from the findings is that direct links exist between SES, income, gender, and healthcare expenditure, albeit not for all types of expenditures. SES has a positive effect on private expenditures, regardless of age group, and income has a positive effect only for the young (aged 20–29) and the adult (aged 30–64) groups. Gender disparity is not found in the young age group, probably because the study is on single person households, but it is found in the two older groups; usually men spend less than women do. In addition, subjective assessment of state of health serves as a mediating variable in the relationship between household’s socioeconomic characteristics and its spending patterns on healthcare.

## Main text

As in other OECD countries, the financing of the Israeli health care system relies on a mix of several public and private sources. While the public sources consist of government budget (general taxation) and compulsory health insurance, the private sources are composed of voluntary health insurance premiums and out-of-pocket payments. During the first decade of the 2000s, Israel experienced a continuous upward trend in the proportion of private financing, which reached a peak of 37–38% toward the end of that decade and then it levelled off. The 38% figure is significantly higher than the 26% average for OECD countries. This trend began shortly after the initial implementation of the National Health Insurance (NHI) Law in 1995, under which universal coverage and a comprehensive basket of health care services are guaranteed. Since its legislation, the NHI Law has permitted the sick funds, which are responsible for providing all their members with care in the community and hospital services, to impose co-payments for several medical services included in the public basket, such as medicines and visits to professional doctors. More importantly, the law allows the sick funds to offer their beneficiaries a voluntary health insurance program that anyone is entitled to join and receive the covered services, regardless of his health status, age, and economic situation. Over the years, the array of services included in this voluntary health insurance has expanded, and currently it has a complementary, supplementary, and duplicative role in the Israeli health system. Its policies cover services that are not included in the NHI health care basket, services that are covered by the NHI—but only to a limited extent—and services that are covered by the NHI and can be purchased in the private sector. Such services are provided in the private sector with enhanced choice of provider, faster access, or improved facilities. In 2016, nearly 83% of Israel’s households purchased voluntary health insurance from the sick funds, and 43% owned commercial voluntary health insurance. The prevalence of multiple coverage, which is very high (41% of all households), raises concerns that consumers may be paying twice for overlapping coverage. In addition, most (88%) of those who did not own voluntary sick fund insurance did not have any commercial insurance as well. In 2016, spending on voluntary health insurance accounted for 35% of total private expenditures.

Existing international literature has dealt with the question of what could explain the increasing trend of private financing in several other countries —whether it is the supply shortages and insufficient public health care financing or rather the result of an income-induced demand for supplementary, higher-quality services from the private sector ([2–5, 10]). The prevailing opinion among Israeli academics and health experts is that, in Israel, the public health system, and in particular the public general hospitals, suffer from a shortage of resources, mostly because the budget has not been updated in line with factors such as population growth, aging, and technological advances. Compared to the OECD average, all measures for hospital activities, such as rates of acute care beds and nurses and physicians per capita, indicate that Israeli public hospitals are crowded and function under pressure. Consequently, individuals with voluntary health insurance coverage look to private hospitals in order to access care, and health insurance became the main source of funding for private hospitals’ activities. The main reasons for preferring private hospitals is the possibility of selecting the surgeon, which is not allowed in public hospitals, and shorter waiting times for certain elective procedures. The growing demand for for-profit hospitals and particularly for surgery in turn had an adverse impact on the public sector as loss of revenue for public hospitals, competition for senior physicians’ time and longer waiting times for elective operations.

However, given that health care services are normal goods, as documented in previous research (e.g. [2, 5, 10]), there is room to assume that the growth in real incomes occurring over the last two decades in Israel has contributed—at least to some extent—to the increase in the relative share of private financing in total finance. Higher incomes allow for buying private services, luxurious facilities, and shorter waiting times as well**.**

The model suggested by Tur-Sinai et al. [7] refers to demographic variables such as gender and age and to income and SES as demand-side determinants of private expenditure, but not as supply-side determinants of the public health care system. Possible candidates for supply-side factors at the locality (of residence) level are, for example, distance from the center of the country as well as the availability of public hospital beds, doctors and nurses, and technologically advanced equipment. The inclusion of supply-side factors into the model may help determine the role played by income versus supply-side factors and explore whether there is an increasing substitution of public health services for private ones.

Several studies have examined the determinants of out-of-pocket expenditures in high-income countries, such as the United States [4], Australia [3], and Germany [2], and in low and middle-income countries, such as China [10] and Sri Lanka [6]. Some of the studies highlight the importance of supply side factors next to demand-side factors, whereas others find that income is the key driver and there is little indication of the effect of limited supply of public health care on rising private expenditures. The concern expressed in this literature is that private expenditures impose a significant financial burden on poor households and act as a barrier for healthcare use. If rich people and the middle class increasingly opt out of public health care, thus spending more on private services and purchasing generous voluntary insurance plans, their willingness to pay taxes to finance the public health care might shrink.

Many non-Israeli studies (e.g. [2–5, 10]) explore the various determinants of health, health care utilization, or spending. Among the determinants are demographic and socioeconomic variables at the individual or household levels, such as type of household, income, education, location, and state of health, as well as variables at the community or locality level. The aim of these studies is to explain inequalities in the dependent variable, i.e., health, service utilization, or spending. What we do know suggests that inequalities in health or in spending on health services very largely reflect inequalities in their determinants. An analysis frequently offered in the literature is to assess each determinant’s relative importance to the measured inequality. The results of these analyses indicate that policies aimed at combating health sector inequalities should aim to reduce inequalities in both the quality and availability of health services on the supply side, and in income, knowledge–especially health-specific knowledge— accessibility of health services, etc. on the demand side. Furthermore, health policy-makers should work more closely with other policy-makers and take a wider view of the multidimensional disparities.

Inequity in health financing is likely to have two adverse effects: it adversely affects access to health services, which can lead to greater inequality in health status in the end. It also can adversely affect income distribution. Although the purpose of health financing is not to redistribute income, its impact on the distribution of income is of obvious interest and importance to policy-makers. Most hold that health system payments should be set according to household ability to pay; and from this point of view, health financing should not be linked to utilization. The principle of “ability to pay,” approximated by income or total expenditures for consumption, includes two dimensions: vertical equity (progressivity) and horizontal equity. Thus, it implies that people with higher incomes should pay more, and those deemed equal should be treated equally. Vertical redistribution occurs when healthcare payments are disproportionally related to “ability to pay,” and horizontal redistribution occurs when people with equal “ability to pay” contribute unequally to the health care system.

In a seminal cross-country study of 13 OECD countries, Wagstaff and Van Doorslaer [8, 9] computed Kakwani indices for assessing the redistributive effects the various public and private sources of healthcare finance around the 1990s. The Kakwani index measures progressivity by comparing the distribution of the finance of healthcare among households to the distribution of their income (when in both cases the households are ranked according to their income). If the curves of the two distributions are overlapping (i.e. the relative share of each decile in the finance of healthcare is equal to its share in total income), the finance is proportional, and if the distribution curve of the finance of healthcare lies above that of income, the finance is regressive and vice versa. The findings Wagstaff and Van Doorslaer indicate that public sources as a whole are progressive, whereas total private sources have a regressive impact on income distribution. In general, out-of-pocket payments are the most regressive source of financing. With the exception of the U.S and Switzerland, total finance is more or less proportional to income (slightly progressive or slightly regressive). For Israel, Achdut [1] examined the redistributive effects of health care finance before and after the implementation of the NHI Health Law in 1995, and found that the finance of the national health expenditures was regressive before 1995, but became only slightly progressive, almost proportional to income. A more recent examination for 2013, presented to the Committee for Strengthening the Public Healthcare System (The German Committee), showed that overall the finance of national health expenditures is slightly regressive: public financing is progressive, whereas private financing is markedly regressive. Furthermore, while out-of-pocket payments and premiums for voluntary health insurance provided by the sick funds are quite regressive, payments for commercial insurance premiums are progressive. This is because high-income households spend more on commercial insurance than other households, and their share in total spending on commercial insurance is even greater than their share in total income.

## Conclusions

Finally, there is a broad consensus among Israeli policymakers on the need to reduce the heavy reliance of the health system on private financing. Indeed, since 2015, the government has introduced several reforms aimed at enhancing regulation on the commercial health insurance market, in order to reduce multiple insurance, and at limiting the diversion of patients and physicians from the public to the private system. Especially noteworthy is the full-timers plan that Ministry of Health has initiated, according to which selected physicians in public hospitals would be offered significantly higher pay in return for working additional hours in a public hospital and giving up work in the private sector. The overall objective of this initiative is to strengthen the public health care system by improving its availability and quality. In future studies it will be important to assess the effect of these recent policies on Israel’s reliance on private financing and on the extent of equity in Israeli health care.

## Data Availability

Not applicable.
